# Evaluation of sexual function after obstetric anal sphincter injury: a 12-year follow-up cohort study

**DOI:** 10.1093/sexmed/qfag047

**Published:** 2026-06-22

**Authors:** David Baud, Maud de Rham, Karine Lepigeon, Chahin Achtari, Baptiste Tarasi

**Affiliations:** Materno-Fetal and Obstetric Research Unit, Woman-Mother-Child Department, University Hospital of Lausanne (CHUV), 1011 Lausanne, Switzerland; Materno-Fetal and Obstetric Research Unit, Woman-Mother-Child Department, University Hospital of Lausanne (CHUV), 1011 Lausanne, Switzerland; Materno-Fetal and Obstetric Research Unit, Woman-Mother-Child Department, University Hospital of Lausanne (CHUV), 1011 Lausanne, Switzerland; Materno-Fetal and Obstetric Research Unit, Woman-Mother-Child Department, University Hospital of Lausanne (CHUV), 1011 Lausanne, Switzerland; Materno-Fetal and Obstetric Research Unit, Woman-Mother-Child Department, University Hospital of Lausanne (CHUV), 1011 Lausanne, Switzerland

**Keywords:** sexual function, obstetric anal sphincter injury, perineal lacerations, third- and fourth-degree tear, vaginal delivery

## Abstract

**Introduction:**

Obstetric anal sphincter injuries (OASIS) may lead to pelvic floor complications, including sexual dysfunction. While short- and medium-term studies have reported increased dyspareunia and decreased sexual pleasure, desire, lubrication, and orgasm ability following OASIS, evidence on long-term sexual function remains limited.

**Aims:**

This longitudinal follow-up cohort study aimed to assess sexual function 12 years after vaginal delivery in women with and without OASIS, and to evaluate its evolution over time.

**Methods:**

The questionnaire used in our previous study, including patients’ sociodemographic characteristics and the Female Sexual Function Index (FSFI), was mailed to the same cohort of women previously included in a case-control study. The FSFI is a validated questionnaire assessing female sexual function across 6 domains: desire, arousal, lubrication, orgasm, satisfaction, and pain. Mean FSFI total scores, domain scores, and individual item scores were compared between women with and without OASIS. Changes in sexual function were also evaluated in comparison with the results obtained 6 years after the index delivery.

**Results:**

Completed questionnaires were returned by 52 women in the OASIS group and 141 women in the control group, with an overall participation rate of 79.8%. Mean FSFI total scores and domain scores were similar between women with and without OASIS. However, lubrication during sexual activity for less than half of the time (19.6% vs 8.5%, *P* = .033) and pain during vaginal penetration for about half of the time or more (13.7% vs 5.0%, *P* = .041) were reported more frequently in women with OASIS. In contrast to findings at 6 years, difficulty reaching orgasm was no longer significantly different between groups (10.0% vs 5.0%, *P* = .212). No significant changes were detected over time in individual FSFI items, domain scores, or total score.

**Conclusion:**

Overall sexual function does not appear to differ between women with and without OASIS 12 years after vaginal delivery. However, women with OASIS continue to report more frequent pain during vaginal penetration and reduced lubrication during sexual activity in the long term.

## Introduction

In women, obstetric trauma leading to obstetric anal sphincter injuries (OASIS), also known as third- and fourth-degree perineal lacerations, occurs in 3.8% of vaginal births. Risk factors are multiple and include, among others, nulliparity, advanced maternal age, obesity, fetal macrosomia, occiput posterior presentation, prolonged labor, and instrumental vaginal delivery.^1^

Obstetric anal sphincter injuries may lead to several complications, including sexual dysfunction. While short- and medium-term data on sexual dysfunction following OASIS have been described in the literature, mainly showing an increase in dyspareunia and, in some cases, a decrease in sexual pleasure, desire, lubrication, and orgasm ability,^2^ data on long-term sexual dysfunction and its evolution over time remain scarce. This gap contrasts with the more extensive literature on long-term fecal incontinence after OASIS. Most studies evaluating sexual function have focused on outcomes up to 5-6 years after delivery, and, to our knowledge, only the study by Mous et al.^3^ has provided data beyond 10 years, reporting a persistent increase in dyspareunia after OASIS.

In a previous study, we demonstrated that women with OASIS were more likely than controls to report difficulties reaching orgasm, impaired vaginal lubrication during sexual activity, and pain during vaginal penetration.^4^ The present longitudinal follow-up study evaluated sexual function and its possible changes over time in the same cohort of women, twelve years after the index delivery. The evolution of fecal and urinary incontinence in this cohort has also been investigated and is reported elsewhere.^5^^,^^6^

## Materials and methods

The initial case-control study^4^ was based on the obstetric database of the the Centre Hospitalier Universitaire Vaudois (CHUV) in Lausanne, Switzerland. This registry contains socio-demographic information, pregnancy-related variables, and perinatal outcomes documented at hospital admission and updated after delivery by the attending obstetrical team.

Women aged ≥18 years who had a vaginal delivery of a singleton fetus in cephalic presentation between 1996 and 2006 were eligible for inclusion (*n* = 13 036). Among this population, 1.5% (*n* = 196) sustained an OASIS. All injuries were repaired using the overlap technique, which was the standard procedure in our institution during that period.

A questionnaire assessing current social, demographic, and physical characteristics, including the Female Sexual Function Index (FSFI), was mailed to the 196 women with OASIS and to 588 matched controls with an intact perineum or first- or second-degree perineal lacerations. Matched controls were selected considering age, parity, ethnicity, year of delivery, birth weight, previous cesarean section, mode of vaginal delivery, and episiotomy.

The FSFI assesses female sexual function over the past 4 weeks. It consists of 19 items divided into 6 main domains: desire, arousal, lubrication, orgasm, satisfaction, and pain. Items are rated on a Likert scale from 0 to 6, with higher scores indicating better sexual function. A total score below 25 was defined as potential sexual dysfunction.^7^

Data analysis of the initial study included 66 women with OASIS (third- and fourth-degree perineal lacerations) and 192 matched controls (intact perineum or first- or second-degree perineal lacerations), for a total of 258 women. The mean time between delivery and response to the questionnaires was about 6 years.

Six years after the initial analysis, a follow-up study was conducted involving the same cohort of 258 women. The same questionnaire used in the initial study was sent to the 242 women for whom contact information remained available (16 participants were lost to follow-up). The return of the completed questionnaire was considered informed consent to participate in the study.

Mean FSFI total scores and domain scores were compared between women with and without OASIS. In addition, individual FSFI items were explored descriptively to identify whether specific symptoms within each domain were more frequently reported in 1 group than in the other group. These item-level analyses were considered exploratory and were not interpreted as independent validated measures of sexual dysfunction.

Pearson’s Chi-squared test and Fisher’s exact test were used for categorical variables. Continuous variables and medians were compared by using the Student’s *t-*test or Wilcoxon test for normally and non-normally distributed data, respectively. Paired comparisons between 6 and 12 years were performed using the Wilcoxon signed-rank test. We estimated a necessary sample size of 47 women with OASIS and 141 controls to achieve an 80% power in detecting a 20% difference with a significance level of 0.05. Anticipating a participation rate of 70%, the sample size expected should meet the objective.

The study was approved by the local institutional review board (CER-VD #101/08).

## Results

Completed questionnaires were returned by 52 of 63 women (82.5%) in the OASIS group and 141 of 179 women (78.8%) in the control group. Three participants did not complete the FSFI questionnaire and were not included in the analyses. No participant reported the absence of sexual activity during the period assessed by the FSFI. The FSFI global score at the time of the first study did not show any significant differences between respondents and non-respondents.

The mean age was 42.0 ± 5.7 years (30-54) in the OASIS group and 41.9 ± 5.4 years (30-55) in the control group. Sociodemographic characteristics at the time of questionnaire response, as well as obstetrical data (including induction of labor, prolonged second stage of labor, mediolateral episiotomy, persistent occiput posterior position, instrumental delivery, and birth >90th percentile), were similar between women with and without OASIS. At 12 years after the index delivery, menopausal status was similarly distributed between the 2 groups: 7.8% of women with OASIS and 4.3% of controls were menopausal (*P* = .461). Similarly, marital status was comparable, with 86.5% and 84.6% of women being married in the OASIS and control groups, respectively (*P* = .739).

The mean FSFI scores were similar between both groups (*P* = .723) as well as the 6 sub-scores. As observed after 6 years, lubrication during sexual activity for less than half of the time and pain during vaginal penetration for about half of the time or more were significantly more frequently reported in the OASIS group (19.6 vs 8.5, *P* = .033 and 13.7 vs 5.0, *P* = .041, respectively). In contrast to findings at 6 years, there was no longer a significant difference in difficulty reaching orgasm (10.0 vs 5.0, *P* = .212) (Table 1).

**Table 1:** Female Sexual Function Index (FSFI). Continuous FSFI domain and total scores are reported as mean ± SD, whereas dichotomized individual FSFI items are reported as proportions with relative risks and 95% confidence intervals.

**FSFI results**					
**Questionnaire items**	**OASIS (*n* = 52) %**	**Controls (*n* = 141) %**	***P* value**	**RR**	**95% CI**
Sexual desire for half of the time or less	17.7	18	.957	0.98	0.49-1.96
Low level of sexual desire	17.7	20.6	.654	0.86	0.44-1.69
Score “Desire” (1.2-6)	4.0 ± 1.1	3.9 ± 1.0	.615		
Arousal during sexual activity for half of the time or less	15.7	11.4	.422	1.38	0.63-3.03
Low level of arousal during sexual activity	11.8	9.9	.713	1.18	0.48-2.92
Low confidence about becoming sexually aroused during sexual activity	11.8	8.6	.504	1.37	0.54-3.47
Satisfied with arousal during sexual activity for less than half of the time	13.7	9.2	.367	1.49	0.63-3.52
Score “Arousal” (0-6)	4.5 ± 1.2	4.47 ± 1.14	.725		
Lubrication during sexual activity for less than half of the time	19.6	8.5	.033	2.30	1.06-5.00
Difficulty becoming lubricated during sexual activity	9.8	5.7	.314	1.73	0.59-5.04
Maintain lubrication until completion of sexual activity for less than half of the time	17.7	10.1	.156	1.75	0.81-3.80
Difficulty maintaining lubrication until completion of sexual activity	6	8	.763	0.75	0.22-2.59
Score “Lubrication” (0-6)	4.9 ± 1.5	5.0 ± 1.2	.392		
Reach orgasm for less than half of the time	18	15	.618	1.20	0.59-2.44
Reaching orgasm difficult	10	5	.212	2.00	0.66-6.02
Moderately dissatisfied with ability to reach orgasm	24	14.3	.115	1.68	0.89-3.18
Score “Orgasm” (0-6)	4.5 ± 1.5	4.8 ± 1.3	.230		
Moderately satisfied with amount of emotional closeness during sexual activity	26	15.7	.107	1.65	0.90-3.03
Moderately dissatisfied about the sexual relationship	18	12.3	.319	1.46	0.70-3.06
Moderately dissatisfied about overall sexual life	20	14.3	.342	1.40	0.70-2.78
Score “Satisfaction” (0-6)	4.4 ± 1.5	4.4 ± 1.3	.857		
Pain during vaginal penetration for about half of the time or more	13.7	5	.041	2.75	1.01-7.44
Pain following vaginal penetration for more than half of the time	5.9	5	.728	1.18	0.32-4.38
High level of pain during or following vaginal penetration	7.8	7.4	1.000	1.07	0.35-3.25
Score “Pain” (0-6)	5.2 ± 1.5	5.3 ± 1.3	.744		
Total FSFI score (2-36)	27.5	27.9	.723		

Abbreviations: FSFI, Female Sexual Function Index; OASIS, obstetric anal sphincter injuries; 95% CI, 95% confidence interval.

**Figure 1:**
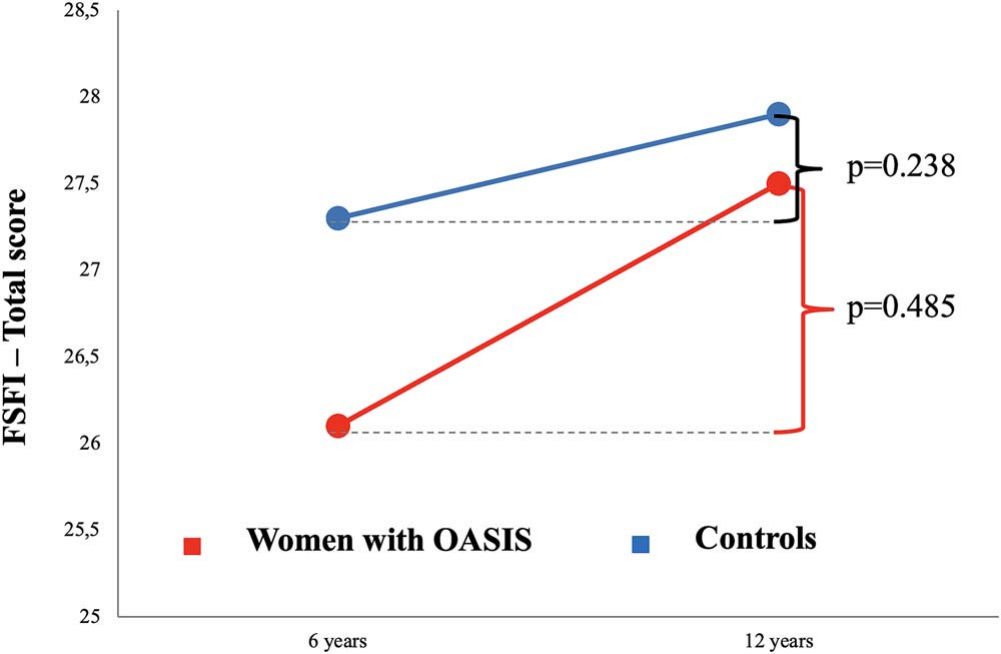
Evolution of sexual function index.

A slight, non-significant improvement in the overall FSFI total score was observed between 6-12 years (Figure 1). However, no statistically significant changes were detected in any individual FSFI items or sub-scores, either in the overall population or within each group separately (data not shown).

## Discussion

Within our cohort, OASIS was identified in 1.5% of women who had a vaginal delivery of a singleton fetus in a cephalic presentation. Our findings indicate that, in the long term, 12 years after the index delivery, women who experienced OASIS during childbirth were more likely to report difficulties with lubrication during sexual activity and pain during vaginal penetration than women without OASIS, contributing additional data to the limited evidence currently available. These associations should be interpreted cautiously, as the confidence intervals were wide and their lower bounds were close to the null. Moreover, because the FSFI is primarily validated for interpretation through total and domain scores, findings based on individual items should be considered descriptive only.

The longitudinal analysis suggests that overall sexual function remained broadly stable between 6 and 12 years after delivery. Therefore, the slight increase in FSFI total score should not be interpreted as evidence of significant improvement.

The persistence of dyspareunia and lubrication difficulties after OASIS may be explained by several mechanisms, although these were not directly assessed in our study. Perineal fibrosis, scar rigidity, persistent perineal sensitivity, residual anatomical defects after sphincter repair, and pelvic floor muscle dysfunction may contribute to long-term pain during vaginal penetration. Lubrication difficulties may also be related to chronic pain, anticipatory anxiety, and avoidance of sexual activity, which can interfere with normal sexual arousal.^3^^,^^8^^,^^9^

During both short- and long-term follow-up of women with a history of OASIS, gynecologists should actively assess the presence of sexual dysfunction. Management options such as vaginal estrogens, lubricants, and individualized counseling or support may be considered when appropriate.^10^

The long-term follow-up of this cohort, allowing evaluation twelve years after the index delivery, represents an important strength of the present study and contributes to the limited long-term data available in the literature. The high response rate also strengthens the reliability of our findings.

However, several limitations should be acknowledged. First, the number of participants remains moderate and although controls were initially matched for several obstetric and demographic characteristics, no additional multivariable adjustment was performed for other potential confounders in the present follow-up analysis.

While lubrication difficulties and pain during vaginal penetration were more frequently reported in women with OASIS, these findings should be interpreted cautiously. Menopause was reported by 9.3% of women with sexual dysfunction (FSFI < 25), compared with 3.8% of women without sexual dysfunction (FSFI ≥ 25) (*P* = .227). However, the limited number of menopausal women prevented robust adjustment for this variable. Age-related hormonal changes may therefore have contributed to these symptoms.

Finally, baseline symptoms were not assessed through questionnaires, which limits our ability to determine whether the reported symptoms were already present before the index delivery.

## Conclusion

Twelve years after childbirth, overall sexual function appears comparable between women with and without OASIS. However, women with OASIS continue in the long term to report more frequent pain during vaginal penetration and difficulty with vaginal lubrication during sexual activity.
